# Thermal Decomposition Behavior of Prussian Blue in Various Conditions

**DOI:** 10.3390/ma14051151

**Published:** 2021-03-01

**Authors:** Durga Parajuli, Hisashi Tanaka, Koji Sakurai, Yukiya Hakuta, Tohru Kawamoto

**Affiliations:** Nanomaterials Research Institute, National Institute of Advanced Industrial Science and Technology (AIST), 1-1-1 Higashi, Tsukuba 305-8565, Japan; hisashi.tanaka@aist.go.jp (H.T.); koji-sakurai@aist.go.jp (K.S.); y-hakuta@aist.go.jp (Y.H.)

**Keywords:** Prussian blue, radioactive cesium, decontamination, thermal decomposition, exothermic oxidation

## Abstract

Prussian blue analogs (PBA) are widely studied for radioactive cesium decontamination. However, there are fewer works related to their post use storage. Considering the oxidative stabilization of the material after the selective uptake of Cs, the thermogravimetric properties in powder and bead form, with various Cs and other alkali metal ions adsorbed, and various heating rates were studied. TG-DTA taken in dry air condition shows an exothermic decomposition at ~270 °C. This temperature varied with the heating rate, mass, and the proportion of adsorbed ions. The best condition for complete oxidation of Prussian blue (PB) is found to be a gradual oxidative decomposition by heating in the temperature range of 200–220 °C until the total mass is decreased by >35%. After this, the temperature could be safely increased to >300 °C for the complete oxidative decomposition of PB that formed iron oxide and salt of the adsorbed Cs. A pilot scale test conducted using the radioactive Cs adsorbed Prussian blue microbeads (PB-b) confirmed that no Cs was released in the effluent air during the process.

## 1. Introduction

Ferric hexacyanoferrate (Fe-HCF), commonly known as Prussian blue (PB), is an inorganic complex, which is famous for its peculiar cesium (Cs) selectivity [1,2,3]. PB forms an open cage structure that possesses a typical zeolite characteristic, an adjective given to any molecule capable of trapping other ions within its lattice cavities [2,4]. Interestingly, PB, different from the common zeolite materials, possesses unique selectivity for the alkali metal ions, especially the Cs-ion [5,6,7,8,9,10]. Due to this structural coincidence, PB could be considered the ultimate Cs-decontaminant. Based on the coordination concept of it, several transition metal hexacyanoferrate derivatives known in general as Prussian blue analogs (PBA) are synthesized and characterized for Cs removal and several other applications [7,8,9,10,11,12,13,14,15,16,17,18,19]. Interestingly, irrespective of the variation in their chemical and physical properties, the PBA possess Cs adsorption ability offering a choice for the assured removal of Cs in different systems. From Ni-analog for Cs in alkaline solutions to Cu-analog for the electrochemical removal of Cs or PB for neutral to acidic solutions, no other material shows high Cs selectivity, faster kinetics, and high capacity like the PBA [8,20,21].

Regarding the use of PBA for radioactive Cs decontamination, one of the targets is the long-living ^137^Cs that decays as [5.4% ^137^Cs → ^137^Ba (1.174 MeV β^−^); 94.6% ^137^Cs → ^137m^Ba (0.512 MeV β^−^) → ^137^Ba (0.6617 MeV γ)] [22,23]. Therefore, from the aspect of environmental Cs decontamination, selectivity and the effectiveness in low concentration is the key. When selectivity matters, the use of PBA is the best choice as it offers faster adsorption kinetics, effective decontamination efficiency even in very low concentrations and overall thousands of folds of volume reduction of the contaminated systems [18,19]. The systems like Cs-containing ash, soil, contaminated environmental waters, etc. are mostly rich in several other metal ions [24,25]. In addition, in such systems the concentration of naturally existing ^133^Cs is much higher than its radioisotopes. So, unlike the case of high-level radioactive wastes, even if only Cs is adsorbed to the material, the chances of a tremendous increase in temperature due to excessive enrichment of the radioisotopes is much lower [24]. Even so, there are doubts about the thermal stability of PBA during its prolonged storage after the adsorption of radioactive Cs.

For assured application of the material, a complete scheme for the environmental radioactive Cs decontamination should also include the means for the safest possible storage of the used adsorbent. For this, thermal oxidation of PBA is considered. As the PBA undergo exothermic oxidation upon heating in the presence of air, the thermogravimetric behavior of PB, before and after the adsorption of Cs from different solutions, is studied in detail. Precautions required for the controlled decomposition for avoiding the exothermic temperature surge, the products, and the most relevant scheme drawn based on the outcomes are summarized in this paper.

## 2. Materials and Methods

### 2.1. Material and Methods

PB nanoparticle (8.8 nm primary size) spray-dried to about 60 μm composite powder was supplied by Kanto Chemicals [18]. This water-insoluble material with the tentative composition, as prepared, of Fe^III^[Fe^II^(CN)_6_]_0.75_·5.03H_2_O was used for understanding the basic adsorption and thermogravimetric properties. The Prussian blue microbeads (PB-b) of 80% PB solidified with 20% calcium alginate was used for the scaled-up decontamination test in the pilot plant. For this, first, the PB slurry was prepared by mixing the analytical grade Fe(NO_3_)_3_·9H_2_O, Na_4_Fe(CN)_6_·10H_2_O and sodium alginate, followed by dropping it into the CaCl_2_ solution [8]. 

### 2.2. Characterization Methods

TG 8120 Thermo Plus EvoII by Rigaku Corporation (Tokyo, Japan) was used for the TG-DTA analysis. About 10 mg samples, unless stated otherwise, were put in an Al or Pt pan and heated at various rates in dry air condition. Perkin Elmer-Anton Par Multiwave 3000 microwave decomposition system (Perkin Elmer, Waltham, MA, USA) was used for the complete decomposition of the adsorbents for the composition analysis. For this, 10 mg of the material was immersed in a mixture of 4 mL concentrated hydrochloric acid and 2 mL nitric acid and treated with microwaves of 500 W, then 800 W for 10 min each. Metal concentrations in all experiments were analyzed using Perkin Elmer NEXION 300D ICP-MS. 5 ppb Indium solution in 2% nitric acid solution; used as the internal standard. BUCHI Glass Oven B-585 Kugelrohr (BUCHI Corporation, New Castle, DE, USA) was used for the well-aerated thermal oxidation of PB or PB-b at various temperatures and masses. Nicolet-iS5 spectrometer (Thermo Fisher Scientific, Waltham, MA, USA) in ATR mode was used for taking the FT-IR profiles. Rigaku RINT-Ultima III (Cu Kα radiation, 40 kV, 30 mA, (Rigaku, Tokyo, Japan) was used for taking the powder x-ray diffraction profile. Mossbauer spectra were taken by outsourcing to the Toray Research Center, Inc. The profiles were recorded in the transmission mode taking 50 mg of the material in the sampling paper at room temperature and normal pressure; ^57^Co/Rh matrix (1.85 GBq) was the source. The transition between the ground and the lowest excited states of ^57^Fe at 14.4 keV energy was used for observation.

### 2.3. Adsorption Test

The adsorbent materials (PB and PB-b) were loaded with the various amounts of Cs from the CsNO_3_ solution prepared either in pure water (Milli-Q integral 3, Millipore, Burlington, MA, USA) or in the tree bark incineration fly ash washed extract, according to the method explained in the previous publication [24]. In short, the extract was obtained by passing pure water into a column packed with the fly ash. An aliquot of concentrated hydrochloric acid was added until the solution reached the near neutral pH. PB was mainly studied for the lab-scale test. The pilot plant test was carried out using columns packed with PB-b. In the batch, the materials after the adsorption test were oven-dried at 60 °C for characterization purposes. Columns after the flow adsorption test were air-dried using the rotating oven at 180 °C before increasing the temperature required for the oxidative decomposition. The amount of Cs (and other alkali metals, where indicated) adsorbed onto PB or PB-b was measured by the microwave-assisted solubilization of the adsorbent followed by quantitative analysis using ICP-MS.

### 2.4. Thermal Oxidation of PB and PB-b

Thermogravimetric properties of the materials with Cs adsorbed from pure water solution or an ash washed water was analyzed based on the heating rate and the amount of adsorbed Cs. For this, the material mass was maintained at 10 mg. The heating rate varied from 2 to 40 °C/min. Dry air was passed in all the tests. The heating experiments were first carried out taking the given mass of PB in a porcelain pan or glass watch. In this phase, no additional air was supplied into the oven during heating. The temperature was tracked at different points of the oven. For the mass 1 g or higher, the material was packed in hard-glass columns with two side outlets and heated using the rotating oven (BUCHI Glass Oven B-585 Kugelrohr). In this case, room air was continuously passed into the column. For the pilot-scale test, the adsorbent was packed in a stainless steel column.

### 2.5. Pilot Plant Test

The test was carried out in the month of February (daytime temperature ~10 °C) at the pilot-scale facility established for the cesium radioisotopes (r-Cs) contaminated waste incineration and decontamination purpose. The process of obtaining the fly-ash from the contaminated sources is explained in our previous publication [24]. The ash was washed with water and the extract was passed to two columns packed with 130 g each of PB-b [18]. The Cs concentrated columns were first heated at 180 °C for 1 h followed by heating at 220 °C for 4 h and finally at 300 °C for 10 h. The possible release of r-Cs in the effluent air was continuously tracked in the air outlet by passing the air through cold-water traps. The concentration in the trap solutions was also analyzed. Total mass reduction and the oxidation of PB-b into iron oxides was confirmed by the weight difference.

## 3. Results and Discussion

### 3.1. Thermogravimetric Properties of PB

**Figure 1:**
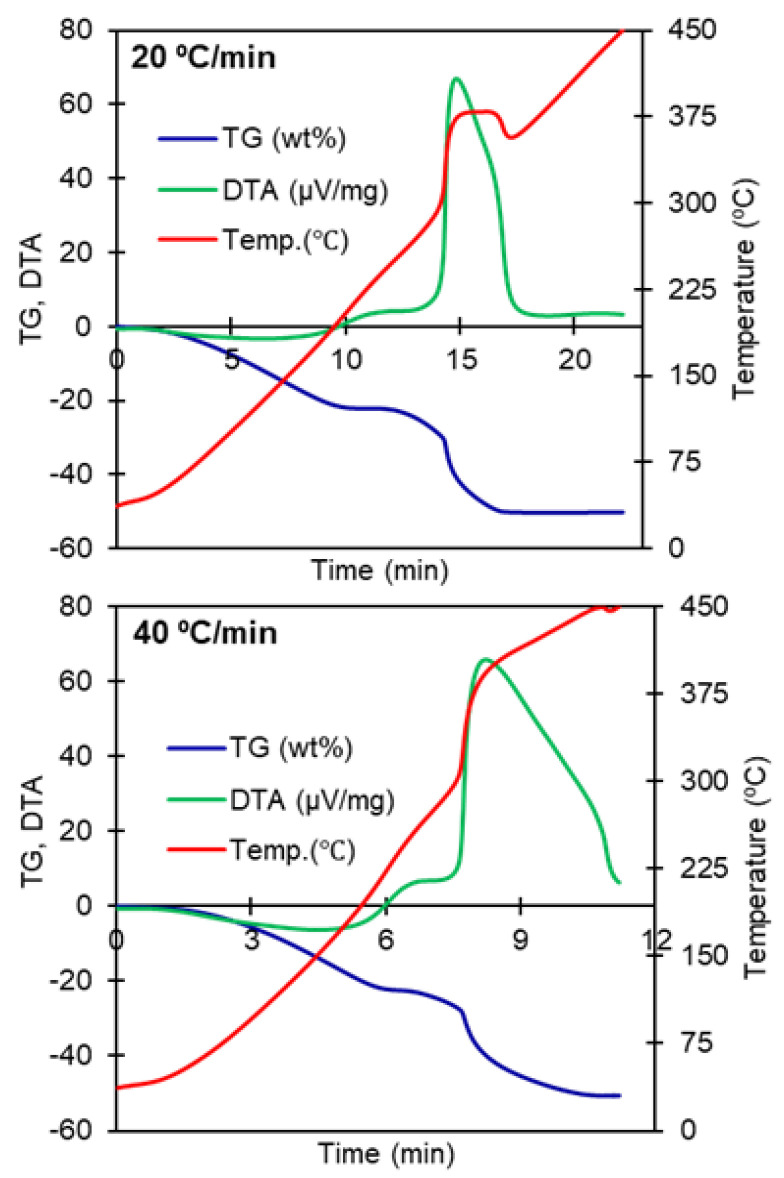
TG-DTA profiles of Prussian blue (PB) at various heating rates. Condition: dry air, mass: 10 mg. Platinum pan. TG (wt.%); DTA (µV/mg).

The purpose of the current study is to understand the oxidative phase change of PB. The profiles were first compared at different heating rates in dry air conditions. As shown in Figure 1, for 10 mg of PB, at 2 °C/min and 5 °C/min heating, the mass loss occurred in three distinct steps. Among these, the first step accounts for water loss, the second being the gradual loss of CN, and the final loss corresponds to the decomposition of the material to iron oxide [26]. When the rate was increased to 10 °C/min and higher, the temperature surge during the exothermic oxidation became distinct and it got uncontrollable at the rate of 40 °C/min. The shape of the DTA profiles explains the impact of fast heating. PB being a mixed oxidation state iron compound, the third exothermic peak, which is assigned for the formation of iron oxide (as discussed in Section 3.5), covers the partial or complete oxidation of Fe^II^ to Fe^III^, depending upon the energy scale. As the oxidation state of iron in each product was not individually characterized at this stage, the correlation between the exothermic energy release and the proportion of Fe^III^ could not be correlated.

The most important information obtained from these five profiles is that if the heating occurs gradually, oxidation of PB takes place very slowly, causing no uncontrollable temperature rise. However, in larger mass, it is difficult to predict the actual temperature profile.

### 3.2. Influence of the Presence of Alkali Metal Ions on the Thermogravimetric Properties of PB

The results shown in Figure 1 suggest that the exothermic decomposition of PB takes place when the temperature is >250 °C. However, when the material is heated very slowly, it may start from a bit lower temperature as well. These results are for 10 mg of the material in a constant flow of dry air and the results are for the material free of Cs and any other cations. When the material used for the Cs uptake from the real decontamination system is stored in mass, what happens? The system of concern is r-Cs loaded PBA packed in a closed shell. This means the material certainly consists of all the other ions, which get trapped with the material during the decontamination process. Therefore, the TG-DTA profile was compared taking PB loaded with Cs from pure water and the ash-extract solution. Due to the radioisotope handling regulation, PB loaded with r-Cs could not be analyzed at this stage.

**Figure 2:**
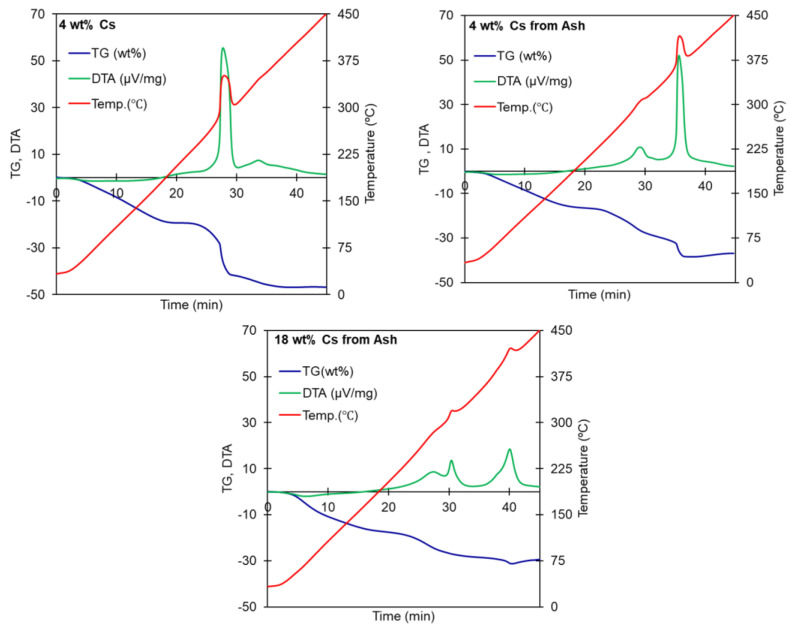
TG-DTA profiles of PB with Cs adsorbed from pure water or the ash extract solutions. Condition: dry air, mass: 10 mg, rate: 10 °C/min, platinum pan. TG (wt.%), DTA (µV/mg).

In order to know the influence of Cs adsorption on the thermogravimetric properties from water that is not pure, the TG-DTAs of PB samples with 4 wt.% Cs adsorbed from pure water and 4 or 18 wt.% Cs adsorbed from the ash washed solution were compared. cesium adsorbed Prussian blue (PB-Cs) with 4 wt.% Cs adsorbed from the ash extract solution contained about 6 wt.% K, whereas that with 18 wt.% Cs contained about 2.5 wt.% K. This accounts for the total of about 10 wt.% and 20.5 wt.% alkali metals in those materials, respectively. As shown in Figure 2, for 10 °C/min heating in dry air condition, the profile of PB loaded with 4 wt.% Cs, in general, looks similar to that without Cs, as shown in Figure 1. However, the thermogravimetric behavior largely changes when the Cs was adsorbed from ash extract solution. For the same 4 wt.% Cs from the ash extract solution the heights of the two peaks belonging to the exothermic reaction are switched. The major peak observed at around 300 °C now becomes relatively very short and the second broad peak has become the major one, showing a significant temperature rise as well. The reason behind the different profiles for the materials with the same amount of Cs is the presence or absence of other alkali metal ions in the adsorption solution as PBA also adsorbs other alkali metal ions although the selectivity is highest for Cs [1,27]. The difference in the total mass loss between 4 wt.% Cs adsorbed from pure water (~48%) and from the ash washed solution (~40%) is due to the decrease in the effective mass of PB in the sample. With the increase in the amount of adsorbed Cs and other ions, the effective mass of PB taken for the TG-DTA drops, which also leads to a decrease in the energy released during hexacyanoferrate (HCF) oxidation. The second DTA peak with increased energy in this case is assumed to be of the oxidation of iron together with the formation of alkali metal compounds.

For PB with 18 wt.% Cs (plus 2.5 wt.% K) adsorbed from the ash extract solution (it was not possible to load more than 4 wt.% of Cs when in pure water solution), the DTA peaks were flattened. In this case, it should be noted that 20.5 wt.% mass is covered by Cs and K. Thus, PB out of 10 mg sample is at least 20.5 wt.% less (Rb not being analyzed). Therefore, if r-Cs are decontaminated from any solution system consisting of several other monovalent cations, PB adsorbs a much higher amount of Cs first and if there is more space other alkali metal ions like K and Rb also get adsorbed. This leads to a change in the thermogravimetric profiles to a great extent, most obvious being the suppression of the exothermic heat flow during the thermal oxidation. It can be understood as the decrease in the effective mass of the CN unit in the material after adsorption leads to a proportional decrease in the corresponding exothermic energy.

From the results summarized in Figure 1, Figure 2, and Table 1, it can be concluded that the exothermic decomposition of PB takes place at a temperature higher than 200 °C. However, the process is significantly slowed down when the heating takes place slowly. Also, in the presence of the high amount of alkali metal ions, the second exothermic peak becomes the major one with a significant decrease in the total heat release.

### 3.3. Thermal Decomposition of PB-Cs: Studying the Possibility of Partial Decomposition

**Figure 3:**
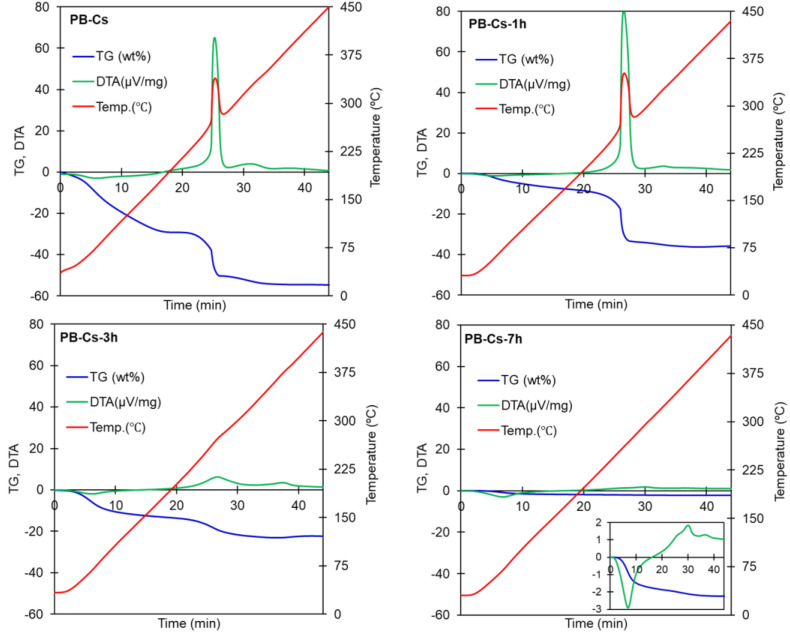
TG-DTA profiles of PB-Cs with 2 wt.% Cs adsorbed from pure water solutions, taken before and after heating stationary at 200 °C for 1 h, 3 h, and 7 h. Condition: dry air, mass: 10 mg, rate: 10 °C/min, platinum pan. Furnace heating: 1 g PB-Cs powder in a watch glass, air enclosed furnace pre-heated to 200 °C.

One more concern about the implementation of PB analogs for the radioactive Cs decontamination is the release of loaded Cs when the material gets decomposed for some reason. Whether it is possible to control the release of Cs by partially oxidizing the material before storage or not is assessed by heating PB-Cs for a different time at different temperatures in a muffle furnace. With 2 wt.% (a loading amount, which can be easily achieved in any solution system) Cs adsorbed from pure water solution when heated at 200 °C for 1, 3, and 7 h with air enclosed in the furnace, an interesting variation could be seen. Comparing the TG-DTA profiles of these heat-treated materials, as shown in Figure 3 for the 1 h sample, although the weight loss is decreased by about 20%, the DTA profile looks similar to additional heat release during the exothermic change. This is because only water weight was lost when PB-Cs was heated for only 1 h, which led to an increase in the effective mass of PB-Cs. However, the 3h heated sample is found to undergo a very smooth conversion with almost no heat surge. The material after heating for 7 h showed a very different profile with the least weight loss; although no significant change in the endothermic dip was observed. Next, the PB-Cs powders before and after heating are mixed with an aliquot of water for checking the Cs releasing tendency. Interestingly, only a trace concentration of Cs was found in the supernatant of the 1 and 3 h samples. For the 7 h sample, nearly all the Cs adsorbed was dissolved into the water.

**Figure 4:**
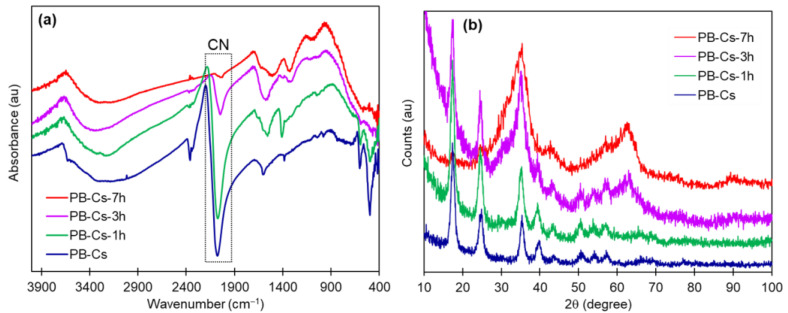
Comparison of FT-IR (**a**) and XRD (**b**) profiles of the PB-Cs samples before and after heating at 200 °C for 1, 3, and 7 h. The *Y*-axis is arbitrary, so only the peak positions should be compared.

The points learned from these observations are: for the sample 1h, only water weight is lost. After heating for 3 h, partial decomposition of the material took place and the 7 h heating leads to nearly complete decomposition. The one with a peculiar observation is 3 h as it did not release Cs to water, although the characteristic heat surge during the exothermic reaction nearly disappeared. The FT-IR and XRD profiles, shown in Figure 4, suggest the presence of characteristic PB peaks. The relative intensities in the FT-IR spectra suggest a sharp decrease in the abundance of –CN, but it has not completely decomposed. The XRD profile also supports the presence of the characteristic PB-peaks. Therefore, the material PB-Cs−3 h still maintains the Cs holding ability. For this reason, Cs was not released to the solution when it was washed with water. On the other hand, PB-Cs−7 h has almost completely lost the structure of PB and hence released the adsorbed Cs quantitatively.

PB is a mixed oxidation state complex typically with 44% of Fe^II^ and 56% Fe^III^. Studies show that the light, temperature, and even change in the solvent system triggers the change in the ratio of Fe^II^ and Fe^III^ [28,29]. This study being about the thermal treatment, gradual oxidation of Fe^II^ is assumed to take place likewise.

**Figure 5:**
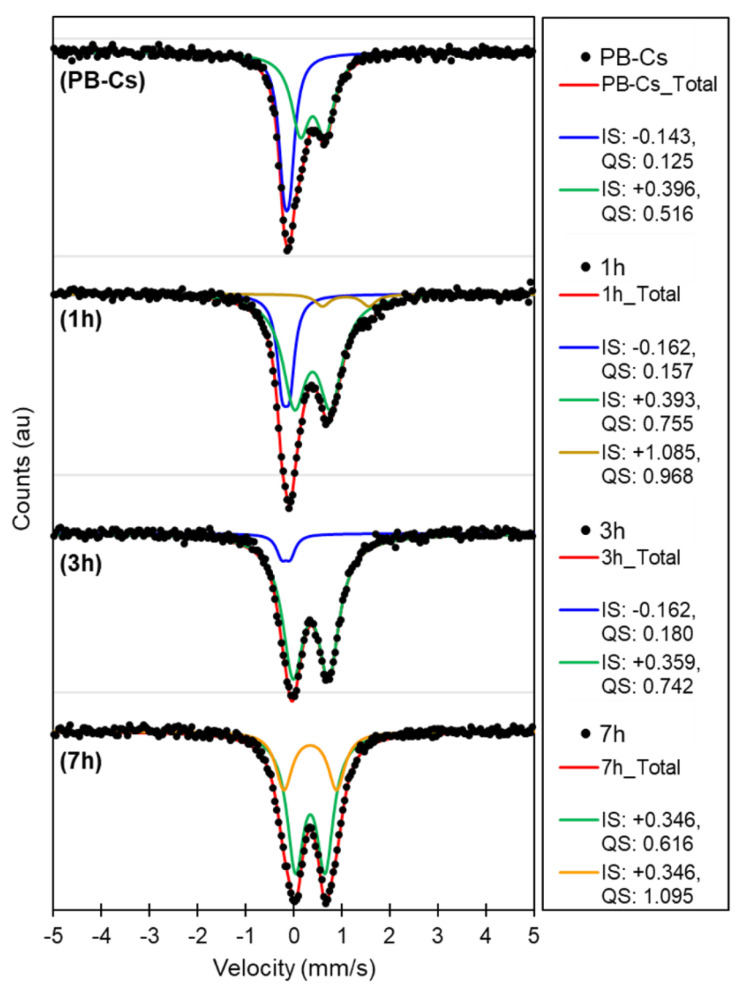
Mossbauer profiles of PB-Cs before and after heating at 200 °C for 1, 3, and 7 h. IS: Isomer shift; QS: Quadrupole shift.

Since the material PB used for this study is the complex of Fe^III^ and [Fe^II^(CN)_6_], variation in the proportions of these states provides information about the material stoichiometry. In the original material, out of the total Fe, 44% is Fe^II^, which has dropped to 29% after 1 h heating and only 7% remains in the 3 h sample, as shown in Table 2. Matching with the XRD profile, the Mossbauer data, in Figure 5, confirms the total decomposition of PB-Cs after heating for 7 h. Generalizing the understanding, profile with negative IS belongs to Fe^II^ and the positive IS belongs to Fe^III^. From before heating PB-Cs to 7 h samples, the proportions of Fe^II^ and Fe^III^ are estimated as 44/56, 29/71, 7/93, and 0/100, respectively. The 44/56 ratio observed in PB-cs is closer to the 43/57 ratio in PB, before adsorption material, calculated from the composition. This means the oxidation of Fe^II^ during the adsorption is unlikely as proton exchange is the key mechanism [4]. This switch in the oxidation states after heating PB is like the past studies [26,29,30]. In Figure 5, a minor third profile was seen in 1 h with IS of +1.085 that could not be identified, so it was neglected. On the other hand, the third profile with the same IS but different quadrupole shift (QS) in sample 7 h is included with that of Fe^III^. Also, the quantitative proportions shown in Table 2 confirm that only a small amount of C and N remain in the 7 h heated sample.

The results discussed suggest two choices for the safer storage of radioactive Cs adsorbed PBA. The TG-DTA data suggests that if the total ferrocyanide is decreased to 15% or so (Fe^II^ in 3 h sample is 16% of that in PB-Cs), the heat surge during the oxidation of the material due to the rise in temperature during the storage could be minimized. By doing this the release of adsorbed Cs can be avoided even if the material comes in contact with water for some inevitable reason. However, controlled thermal decomposition might become more difficult when the system is scaled up. In this sense, the second choice, total oxidation before storage looks more promising. It is well known that PBA highly selectively adsorb Cs. Therefore, for the separation of Cs from a mixture of several ions PBA can be used. In addition, zeolites are known to adsorb a substantial amount of Cs, however, lack the selectivity, especially amongst the alkali metal ions [31,32,33,34,35,36]. Therefore, using PBA for the selective concentration of radioactive Cs followed by its thermal oxidation in a controlled system and finally storage in a shell with zeolite lining can be the safest method. By doing this, not only the highly selective removal of radioactive Cs can be achieved, but also the safe storage can also be accomplished.

### 3.4. Scale-Up Study

For the decontamination of radioactive Cs from the real samples, adsorbent-packed columns were used. Because handling the PB nanoparticle powder in large scale adsorption is challenging, the adsorbent is either loaded to a nonwoven fabric or immobilized as microbeads. In order to know the exothermic phase change during the possible gradual heating of PB, PB-b, microbeads with 80 wt.% PB immobilized with 20 wt.% of calcium alginate are selected for the scale-up the experiment.

**Figure 6:**
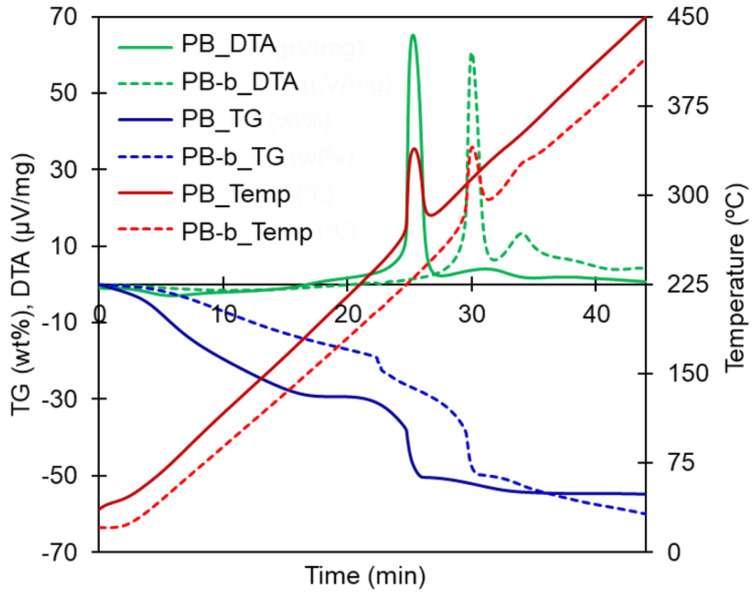
Comparative TG-DTA profiles of PB powder and Prussian blue microbeads (PB-b). Condition: dry air, mass: 10 mg, rate: 10 °C/min, platinum pan.

Based on the results discussed above, it can be assumed that the exothermic decomposition in the presence of air takes place above 200 °C, with some decrease in the heat surge temperature when the mass is increased. Therefore, the thermogravimetric behavior of PB-b is studied before proceeding to the scale-up test. As shown in Figure 6, because the material is now a ϕ1 mm microbead, the phase change took place at a higher temperature (dashed lines). In addition, as the bead form consists of 20 wt.% calcium alginate, the weight loss is a bit higher. Besides these, the TG-DTA profiles and the temperature surge pattern resemble that of PB.

**Scheme 1:**
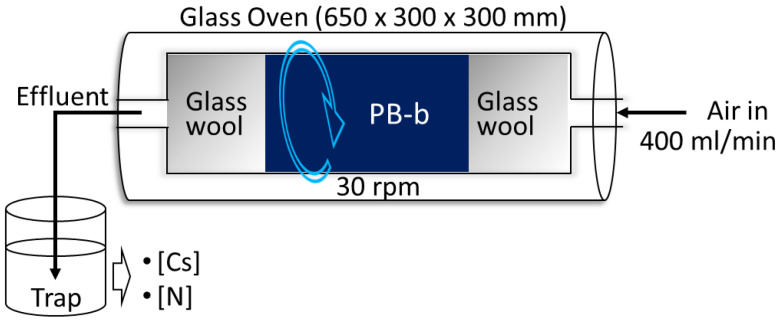
Thermal decomposition of PB-b at a fixed temperature and airflow in a rotating column using a glass oven. The effluent gas was trapped in impingers filled with dilute NaOH solution or only water for solubilizing any gaseous cyanide and Cs carrying particles. Air humidity = 50–55%.

**Figure 7:**
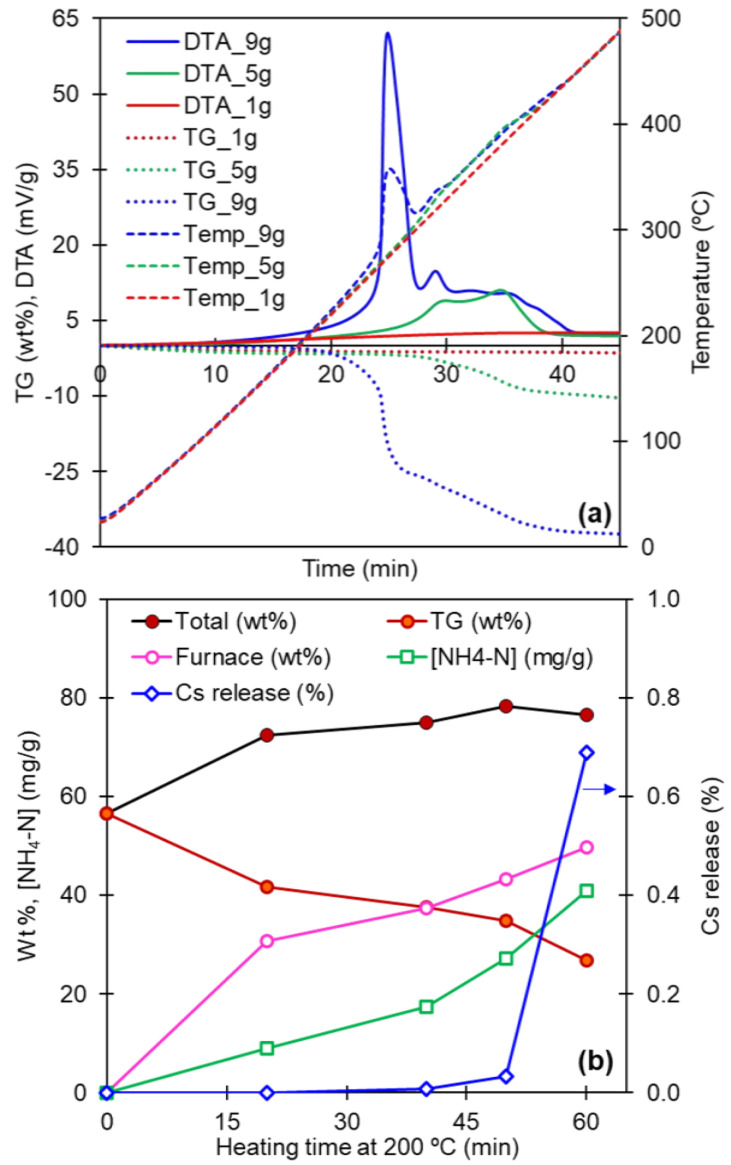
(**a**) TG-DTA profiles of PB-b-Cs taken after heating at 200 °C for 40 min in the oven at different masses. Condition: dry air, mass: 10 mg, rate: 10 °C/min, platinum pan. (**b**) Comparison of the mass loss and the concentration of released N and Cs released during oven heating of 9 g PB-b-Cs at 200 °C for 20, 40, 50, and 60 min. Unfilled circles show the TG mass loss before (0 min) and after the oven heating. Filled circles show the total mass loss after oven heating and TG.

The first scale-up experiment was carried out taking 1, 5, and 9 g of PB-b with 2.5 wt.% adsorbed Cs. The amount of Cs in the adsorbent was estimated by the difference between the concentration of Cs in the feed solution and the residual concentration after adsorption. The heating was carried out using a rotating oven with a continuous flow of air, as shown in Scheme 1. For this, the material was packed in a hard glass container with an opening at both ends. It was then heated at 200 °C for 40 min rotating at 30 rpm and passing the air at the rate of 400 mL/min. After cooling down to room temperature the thermal decomposition status was examined by TG-DTA. As shown in Figure 7a, 1 g fraction showed no visible change in the TG-DTA profile, suggesting the complete decomposition during the oven heating. The 5 g batch showed a changed DTA and a loss of 10 wt.%, but, no temperature surge was observed. On the contrary, the 9 g batch showed a PB-type TG-DTA profile with a significant temperature surge peak. This observation concludes that larger the mass, longer should be the heating time at the given temperature.

In addition to heating the PB material at a controlled temperature in order to avoid the temperature surge, another concern is the release of CN or Cs during the process. So, the 9 g PB-b-Cs packed column was heated at 200 °C for 20 to 60 min and compared for the mass loss and the released species. As shown in Figure 7b, the TG mass loss of PB-b with 2.5 wt.% Cs is about 57%. On the other hand, during oven heating, some 30% weight was lost when heated for 20 min, which increased to 50% after heating for 60 min. The TG profiles of the 20, 40, 50, and 60 min oven heated materials show additional weight loss. However, because of the water regain from the moisture, the TG-loss was higher than expected. This observation concludes that, for 9 g PB-b-Cs, the decomposition was not completed in 1 h heating at 200 °C.

Another important point is the possible presence of CN in the trap solutions. The noteworthy part is that the free CN was either absent or was below the detection limit. However, precipitates of NH_4_HCO_3_ in the tube connecting the effluent gas to the impinger were observed during longer heating. In addition, the concentration of NH_4_-N in the trap solution was found to increase with the decrease in the mass of PB-b. Because it is unlikely that the N_2_ in the air is reduced to NH_4_ when passed through the PB-b column, the only source of “NH_4_-N” is the CN of HCF. As the catalytic conversion of HCN gas to ammonium in the presence of air and moisture is a practical method for getting rid of this toxic gas, a similar mechanism is assumed to be taking place in the present case as well [37]. One more important outcome of the present experiment is that the concentration of Cs in the trap solution is very low, it is expected that it escaped the column along with the fine particles of PB generated once the beads became dry and hot. Using a microfilter in the effluent exit can control the release of this trace of Cs as well.

### 3.5. Complete Oxidation of PB for Ruling out the Temperature rise due to the Presence of HCF

In the scale-up section it is found that heating at 200 °C does not lead to sudden temperature surge, so is considered safe for heating the Cs loaded PB. However, the drawback is that for a larger mass of the material longer heating becomes necessary, which is difficult to estimate. For resolving this issue, the possibility of increasing the temperature after decreasing some mass after heating at 200 °C was considered. By doing this the mass of HCF causing the exothermic oxidation is expected to be controlled. In Figure 7a, for the 5 g batch, there is still the exothermic DTA profile, but the temperature surge peak is missing. Also, the DTA is starting to rise at around 300 °C. Therefore, heating PB-b at 200 °C for a certain time followed by heating at 300 °C for 1 h was considered. Also, the temperature profile at different points of the column and the possible release of Cs due to excessive heat rise was carefully followed.

**Figure 8:**
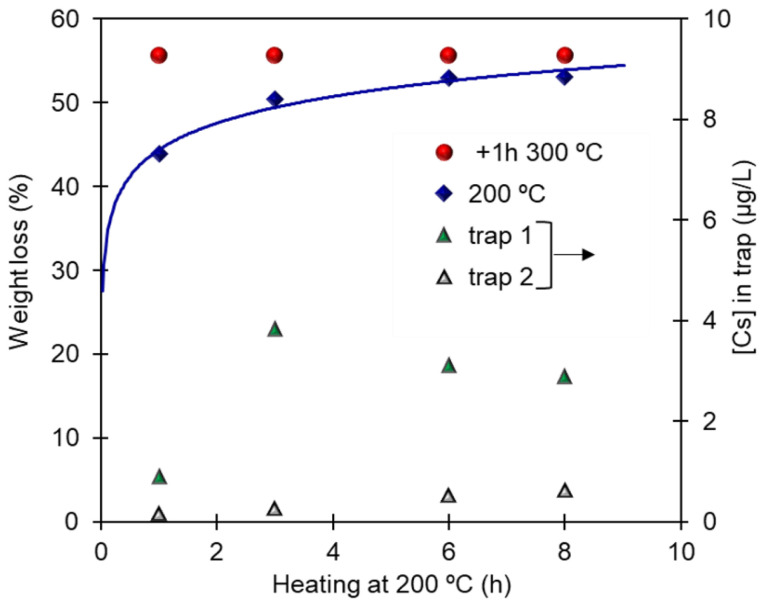
Mass loss of PB-b with 4 wt.% Cs after heating at 200 °C for 1 to 8 h followed by heating at 300 °C for 1 h. The concentration of Cs in trap-1 and trap-2 is for 10 mL of water in the impinger. Mass: 10 g, air flow: 400 mL/min, air temperature: ~20 °C.

For this experiment 10 g of 4 wt.% Cs adsorbed PB-b packed column was heated at 200 °C for 1 to 8 h followed by consecutive heating at 300 °C for 1 h. In all cases room air was passed at the rate of 400 mL/min and the effluent gas was bubbled in two impingers, first with 0.1mol/L NaOH and the second with pure water, in a line. As shown in Figure 8, the mass loss increased with an increase in the heating time. For 10 g of material in the column, no alarming temperature rise was observed while heating at 200 °C. After heating for the given time at 200 °C, the column was weighed in order to determine the mass loss and was again heated at 300 °C for 1 h. For one batch of heating, the same trap solution was used and was finally recovered to measure the concentration of Cs. During 300 °C heating as well, no significant heat surge was observed, suggesting that once a substantial mass is decomposed by heating at the temperature lower than the exothermic oxidation, the heat surge does not take place upon heating at as high as 300 °C.

**Figure 9:**
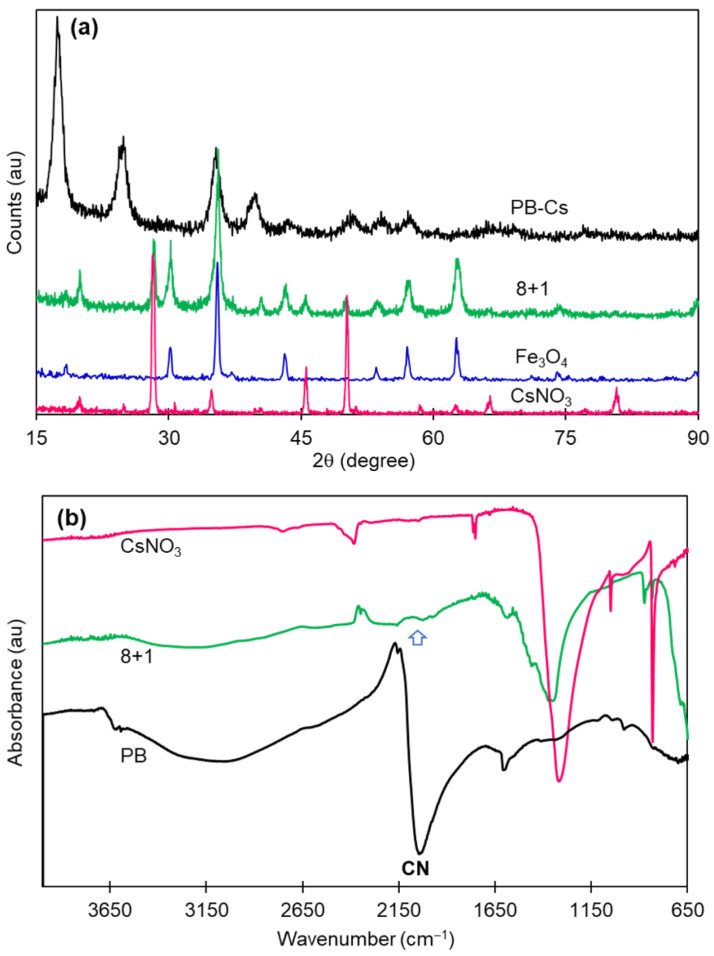
XRD (**a**) and FT-IR (**b**) profiles of PB or PB-Cs before and after the thermal decomposition showing the peaks belonging to the most possible products. 8 + 1 is the material after heating for 8 h at 200 °C followed by 1 h heating at 300 °C in the presence of air.

The products obtained after heating at 200 °C followed by heating at 300 °C for 1 h were characterized by XRD and FT-IR and were found to show resembling profiles. As per Figure 9a, Fe_3_O_4_ seems the closest product [38]. Other studies on the calcination of PB or PBA for various purposes report the formation of iron oxide of various morphologies, depending upon the structure/composition of the starting material and the condition [39,40,41,42,43,44,45]. As mentioned in the previous section, iron in PB, Fe^III^[Fe^II^(CN)_6_]_0.75_, is 43% Fe^II^ and 57% Fe^III^. A closer value of 44% Fe^II^ and 56% Fe^III^ are observed in Mossbauer data of PB-Cs, Table 2. In this sense, the formation of a mixed oxidation state iron compound with 1/3 Fe^II^ and 2/3 Fe^III^, Fe_3_O_4_, can be considered obvious. The decrease in the ratio of Fe^II^ can be correlated to its exothermic partial oxidation.

Regarding the adsorbed Cs, the peaks match with those of CsNO_3_. The FT-IR profile, Figure 9b, confirms the absence of the peaks belonging to the CN, and hence reveal the complete decomposition of PB. It also suggests the presence of the CsNO_3_ kind of material. Yet, a Cs-focused study becomes necessary for confirming the state of the element in the oxidized material. Based on the discussed observations and the characteristic FT-IR and XRD profiles of the final material, it can be presumed that the CN in the PB is oxidized to carbonate, ammonia, and nitrate leading to the formation of ammonium bicarbonate and cesium nitrate. On the other hand, the iron ion undergoes complete oxidation to ferric oxide, as (1). For simplicity, water content in the material and the other alkali metal ions possibly adsorbed during adsorption from ash wash solution are excluded. Fresh air passed during the process is the source of water and oxygen. Presence of one Cs per molecule is the only assumption made for formulating the decomposition equation. The charge balancing role of the hydroxide ion is discussed elsewhere [12].

(1) $$\mathrm{Cs}.{\mathrm{Fe}}_{4}^{\mathrm{III}}{\left[{\mathrm{Fe}}^{\mathrm{II}}{\left(\mathrm{CN}\right)}_{6}\right]}_{3}\left({\mathrm{OH}}^{-}\right)+42H_{2}O+11.75O_{2}\underset{\mathrm{air},\mathrm{heat}}{\to }3.5{\mathrm{Fe}}_{2}O_{3}+{\mathrm{CsNO}}_{3}+17{\mathrm{NH}}_{4}{\mathrm{HCO}}_{3}+{\mathrm{CO}}_{2}$$

### 3.6. Pilot-Scale Test

The environmental radioactive Cs, which are spread outside the designated area due to the nuclear accidents or similar, are categorized as low-level radioactive waste. However, once it is concentrated using the highly selective PB adsorbents, the material needs to be stored safely as per the international regulation set for radioactive waste. For this reason, the glass column was replaced by a stainless column with the capacity of packing ~150 g of PB-b, designed for the pilot-scale test. Before the on-site test, the thermal oxidation experiment was carried out in the lab. However, because of the thickness, the stainless column required longer heating at 200 °C than the glass column. So, the temperature was set to 220 °C for achieving the closer mass loss at the same time. In this case, also, the temperature at the center of the column was closely monitored and no sudden heat surge was observed.

The detail of the pilot-scale test carried out in the Fukushima region on the selective removal of radioactive Cs (along with the naturally existing stable Cs) is discussed in reference [18]. After passing the given volume of the contaminated ash extract, the stainless columns were taken for thermal oxidation. Different from the lab condition, the daytime temperature of the test site on that particular day was about 10 °C. Therefore, the preliminary heating at 220 °C for achieving > 35% mass loss was carried out for 4 h. The observed data are summarized in Table 3. The point to focus on is the total mass loss.

The TG-DTA mass loss of PB-b (before adsorption) is about 57% (Figure 6). According to the data in Figure 2, the mass loss for 4 wt.% Cs adsorbed PB is 47%. This value drops to 37% when the same amount of Cs is adsorbed from the ash extract solution (due to the inclusion of high concentration K present in the extract). When the adsorbed Cs from the ash solution is increased to 18 wt.%, the mass loss is dropped to 30%. However, regarding the data in Table 3, the solution being an ash extract, a significant amount of K is expected to be adsorbed (data not analyzed as the material contained the Cs radioisotopes). As the PB-b consists of 80 wt.% PB and 20 wt.% calcium alginate, the mass loss is a bit higher. So, for the material of concern, 5 h heating at 220 °C and an additional 5 h (different from 1 h in the lab test) heating at 300 °C is assumed to be sufficient for bringing the material to the oxidized forms.

### 3.7. Process Recommendation

It is well accepted that PBA offer the highest selectivity for removing Cs. However, the limiting factor behind its use is the possible release of HCF during the use, which can be easily controlled by the addition of the HCF adsorbent column in the system. Another limiting factor, which we tried to resolve in this study, is the possible decomposition of the material during the storage of the radioactive Cs concentrated PB material. Although the stability related issue is studied in detail and the safety points were already recommended in the 1990s, controlled thermal decomposition of the material prior to storage can be the safest measure for highly assuring storage of the radioisotope concentrated material. Also, as the thermal treatment decreases the material volume to nearly 50% of the starting mass, the remaining space in the PBA packed column can be filled with Cs adsorbing zeolites as a precaution for avoiding the dissolution of Cs in case the material is exposed to water. This study only covered the thermogravimetric behavior of PB. As the oxidative decomposition temperature of each analog varies, the behavior should be studied individually.

## 4. Conclusions

Thermogravimetric data confirm that the exothermic oxidation of PB in the presence of air takes place at >270 °C. The microbeads of PB, PB-b, show a similar TG-DTA profile with some increase in each of the weight loss temperatures. However, the profile is greatly influenced by the heating rate and the amount of alkali metal ions adsorbed (partly because of the decrease in the effective mass of PB). The idea of partial oxidative decomposition for decreasing the mass of the HCF ligand by keeping the Cs holding ability seems challenging, though it sounds very interesting. Heating PB below 180 °C released mostly the water only. It was found that for initiating the gradual oxidative decomposition of PB, heating at >200 °C is required. A most important point to be noted is that the temperature must be kept < 250°C for preventing the material undergoing abrupt oxidative reaction leading to excessive heat surge, which may result in the vaporization of the adsorbed Cs. This vigorous oxidative heat-surge could be controlled by heating the material at a moderate temperature of 200–220 °C until > 35% mass loss is achieved. Afterward, the temperature could be raised to 300 °C at which the material undergoes complete oxidation. The end products of the oxidative decomposition of Cs adsorbed material are found to be iron oxide and cesium nitrate like compounds. The results show a trend of slight variation in the decomposition time with the atmospheric temperature; specific study for the given condition is suggested.

## Figures and Tables

**Table 1:** Variation in the TG-DTA behavior of PB and cesium adsorbed Prussian blue (PB-Cs) in different conditions.

Material	Heating Rate (°C/min)	wt loss (%) Until 450 °C	Temperature Surge	Corresponding DTA Peaks
PB (before adsorption)	2	55.7	not observed	227 °C (low energy broad)
	5	55	twice, small	271 and 353 °C (low energy twin peaks)
	10	50.4	once, high, sharp	281 °C (major, sharp) 320 °C (minor)
	20	50.2	once, high, broad	293 to 360 °C (major, broad)
	40	51	once, high, continued	298 to 450 °C (major, broad)
PB-Cs (heating rate = 10 °C/min)	4 wt.% Cs, pure water solution	47	once, high, sharp	288 °C (major) 325 °C (minor)
	4 wt.% Cs, ash extract	37	twice, small then sharp	292 °C (minor) 360 °C (major)
	18 wt.% Cs, ash extract	29.6	twice, small	315 °C and 360 °C (low energy peaks)

**Table 2:** Data showing the change in the abundance of CN and the oxidation states of Fe in PB-Cs before and after heating at 200 °C for 1, 3, and 7 h.

Values in %	PB-Cs	PB-Cs-1 h	PB-Cs-3 h	PB-Cs-7 h
Data from TG-DTA				
Water	29	8	12	17
PB-Cs except water	71	92	88	83
Weight change after heating				
Wt loss		32.2	46.5	51.1
Loss except water		3.2	17.5	22.1
Forms of Fe calculated from Mossbauer spectra				
Fe^II^	44	29	7	0
Fe^III^	56	71	93	100
CHN analysis				
N in total wt	19.6	21.1	7.31	1.11
N except water	27.6	22.9	8.31	1.34
N released during heating		17.0	69.9	95.1
C in total wt	17.1	16.6	6.6	1.68
C except water	24.1	18.0	7.56	2.02
C released during heating		25.3	68.6	91.6

**Table 3:** Mass loss of cesium adsorbed Prussian blue microbeads (PB-b-Cs) during heating in the pilot scale.

Heating Step	Heating Time (h)	PB-b Mass (g)	Total Mass Loss (%)
Initial	0	129.7	-
220 °C	4	79.9	38.4
300 °C_1st	5	70.6	45.6
300 °C_2nd	5	68.6	47.1
300 °C_3rd	5	67.4	48.0

## Data Availability

The authors confirm that the data supporting the findings of this study are available within the article.

## Abbreviations

HCF	Hexacyanoferrate
PB	Prussian blue
PBA	Prussian blue analogs
PB-Cs	Cesium adsorbed Prussian blue
PB-b	Prussian blue microbead
PB-b-Cs	Cesium adsorbed Prussian blue microbead
r-Cs	Cesium radioisotopes
TG-DTA	Thermogravimetric – Differential Thermal Analysis
